# Early mobilisation by head-up tilt with stepping versus standard care after severe traumatic brain injury – Protocol for a randomised clinical feasibility trial

**DOI:** 10.1186/s13063-018-3004-x

**Published:** 2018-11-08

**Authors:** Christian Gunge Riberholt, Jane Lindschou, Christian Gluud, Jesper Mehlsen, Kirsten Møller

**Affiliations:** 1 Department of Neurorehabilitation/TBI unit, Rigshospitalet, University of Copenhagen, Kettegard Alle 30, 2650 Hvidovre, Denmark; 2 Copenhagen Trial Unit, Centre for Clinical Intervention Research, Department 7812, Rigshospitalet, Copenhagen University Hospital, Blegdamsvej 9, DK-2100 Copenhagen, Denmark; 3 Syncope Centre, Department of Cardiology, Bispebjerg & Frederiksberg Hospital, University of Copenhagen, Nordre Fasanvej 57, 2000 Frederiksberg, Denmark; 4 Department of Neuroanaesthesiology, Rigshospitalet, University of Copenhagen, Blegdamsvej 9, 2100 København Ø, Denmark

**Keywords:** Brain injury, Randomised feasibility trial, Cerebral autoregulation of blood flow, Rehabilitation, Tilt-table therapy

## Abstract

**Background:**

Intensive rehabilitation of patients with severe traumatic brain injury is generally applied in the subacute stages of the hospital stay. Few studies have assessed the association between early and intensive physical rehabilitation and functional outcomes. The aim of this trial is to assess the feasibility of an intensive physical rehabilitation intervention focusing on mobilisation to the upright position, starting as early as clinically possible versus standard care in the intensive care unit. The feasibility study is intended to inform a subsequent randomised clinical trial that will investigate benefits and harms of the intervention.

**Methods:**

This randomised clinical feasibility trial with a follow-up period of 1 year will use blinded outcome assessors for the Coma Recovery Scale–Revised. A maximum of 60 patients admitted to the neurointensive care unit at Rigshospitalet, Denmark, with traumatic brain injury (age of at least 18 years), a low level of consciousness, and stable intracranial pressure will be included in the trial. Patients will be randomly assigned to experimental intervention versus standard care (1:1) stratified according to their Glasgow Coma Score. The intervention group will receive daily mobilisation in a tilt table with an integrated stepping device (ERIGO^®^). Feasibility is declared if more than 60% (the lower 95% confidence interval of the proportion) of eligible patients are included in the trial and more than 52% (the lower 95% confidence interval of the proportion) of patients in the intervention group receive more than 60% of the planned interventions. Safety is assessed by the occurrence of adverse events and adverse reactions. Exploratory clinical outcomes consist of cerebral haemodynamics (blood flow velocity and pressure autoregulation) and baroreceptor sensitivity in the early phase as well as functional outcomes (Coma Recovery Scale–Revised, Early Functional Ability scale, and Functional Independence Measure).

**Discussion:**

Our findings will inform a future, larger-scale randomised clinical trial on early mobilisation using a tilt table early after severe traumatic brain injury.

**Trial registration:**

ClinicalTrials.gov identifier: NCT02924649. Registered on 3 October 2016.

**Electronic supplementary material:**

The online version of this article (10.1186/s13063-018-3004-x) contains supplementary material, which is available to authorized users.

## Background

Patients with severe acquired brain injury (ABI) may benefit from early and intensive rehabilitation, which partly consists of physical exercise [1]. Thus, observational studies have found an association between higher-level physical activities and better final outcome in these patients [1, 2]. However, such exercise poses an orthostatic challenge and requires that the patient be able to compensate for this challenge. Accordingly, for patients with severe ABI and a low level of consciousness, mobilisation to the upright position on a tilt table is an important first step. Several beneficial effects are hypothesised to result from this type of activity. In a recent observational study, we showed that patients with impaired consciousness open their eyes for longer periods of time in the upright compared with the lying position, indicating increased arousal [3]; other authors have confirmed this finding [4, 5] and reported that head-up tilt (HUT) also reduced the risk of ankle contractures (range of motion) and improved lung function [6, 7].

On the other hand, mobilisation to the upright position may trigger haemodynamic problems, including hypotension and syncope, and may also pose a risk of extubation in intubated patients, dislodgement of indwelling catheters, and falls. About 40% of patients with severe ABI have orthostatic intolerance that limits their chance of achieving an upright position [3]. Neither the physiological mechanisms causing orthostatic hypotension nor those that enable recovery from this phenomenon have been thoroughly investigated. Considering analysis of electrocardiography (ECG) signals obtained from ABI patients during HUT, we suggested that impairment of baroreceptor sensitivity may be involved [8]. Whether the impairment is caused by the brain lesion per se or prolonged immobilisation or both remains to be investigated. However, in other patient populations with neurally mediated syncope or orthostatic hypotension, intensive tilt-table training has been shown to be beneficial for regaining neurovascular control [9, 10]. In addition, recent studies including a large number of patients with ABI have found an association between impaired cerebral autoregulation measured the first days after injury and an unfavourable outcome [11, 12]. In line with these results, we have shown impaired autoregulation during HUT in patients with severe ABI as late as 40 days after injury [8]. Thus, it is possible that autoregulation and baroreceptor sensitivity are progressively impaired with prolonged immobilisation and that this further restricts attempts at mobilisation in some of these patients, ultimately leading to an impaired functional outcome.

Even though Andelic et al. found a beneficial effect of early rehabilitation in patients with traumatic brain injury (TBI) in their quasi-randomised trial [13], the net effect of early mobilisation in patients with TBI remains unclear. Also, mobilisation of patients with severe ABI is usually not initiated in the acute stage after injury, during the intensive care stay, but rather at a later, subacute stage (weeks), after stabilisation and transfer for rehabilitation [14]. A recent small study conducted in four patients with acute severe TBI and disorders of consciousness suggested that early mobilisation is feasible and safe using a tilt table with integrated stepping that increases the venous return of blood to the heart [15] but these data warrant replication in larger studies.

In February 2017, we conducted a thorough search of the literature in relevant databases (MEDLINE, CINAHL, EMBASE, CENTRAL, and Web of Science) on early out-of-bed mobilisation in patients with TBI by using Medical Subject Headings (MeSH) terms (brain injuries, traumatic AND rehabilitation). The search showed that no randomised trials have yet been performed in this field.

Therefore, we wish to assess the feasibility of an early HUT protocol in patients with severe TBI, in terms not only of the number of patients who are successfully mobilised but also of the number of adverse events (AEs) and adverse reactions (ARs). In exploratory analyses, we will assess clinical outcomes at 3 months and 1 year. Furthermore, we wish to explore physiological variables during ongoing mobilisation in the early phase and their possible association with the patients’ clinical outcome. Finally, as an exploratory part of this trial, we wish to investigate the occurrence and time to recovery of orthostatic tolerance and cerebral autoregulation in patients with severe TBI who receive early and intense mobilisation and their relation to the functional outcome.

## Methods/Design

**Fig. 1:**
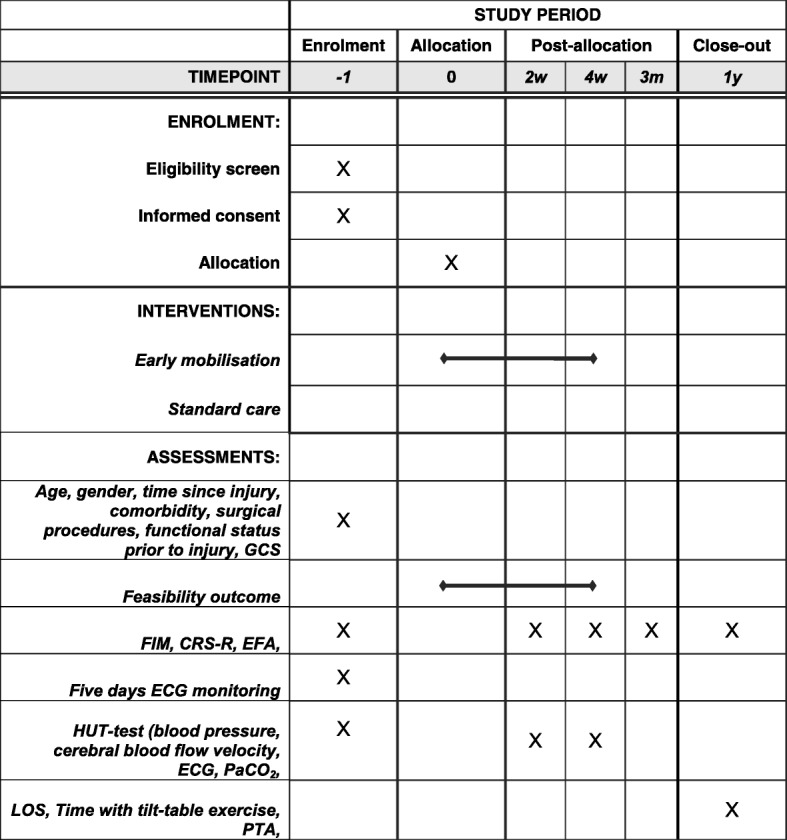
SPIRIT (Standard Protocol Items: Recommendations for Interventional Trials) table of enrollment, intervention, and assessments

This trial is a randomised clinical feasibility trial comparing an early HUT protocol versus standard care in a neurointensive care unit (NICU) and a specialised neurorehabilitation department. The protocol was developed in accordance with the guidelines and checklists for Standard Protocol Items: Recommendations for Interventional Trials (SPIRIT) (Additional file 1) [16]. Results will be reported as stated in the Consolidated Standards of Reporting Trials (CONSORT) statement [17]. Randomisation will be conducted centrally by the Copenhagen Trial Unit using a web-based randomisation system. The allocation sequence will be computer-generated using block sizes of varying length concealed for the investigators. The allocation ratio is 1:1. Because consciousness measured by the Glasgow Coma Scale (GCS) score can be partly a predictor of outcome [18], the allocation sequence will be stratified for GCS score at the time of inclusion (3–6 points compared with 7–10 points). All included patients will be followed from inclusion until 1 year after injury. All baseline assessments will be conducted before randomisation and start of intervention (time point − 1). Cerebral blood flow autoregulation will be studied after 2 and 4 weeks. Functional assessments will be conducted after 4 weeks, 3 months and 1 year (Fig. 1).

### Blinding

It is not possible to blind the intervention for the treating physical therapists or the participant. However, outcome assessment using the Coma Recovery Scale–Revised (CRS-R) will be conducted by assessors who are blinded to the intervention. Data are entered in a validated Microsoft Excel spreadsheet by the primary investigator (CGR) and will be checked for correctness against the source data by a colleague otherwise not involved in the trial. Furthermore, the person analysing the data will be blinded to the patient’s randomisation, and concealed allocation will be revealed only after all analyses have been completed and two conclusions drawn [19].

### Recruitment and informed consent

Patients admitted to the NICU will be screened for eligibility on a daily basis by the principal investigator (CGR). The nearest relative to the patient acts as proxy (next of kin) and is given written information about participation in the trial. The relative is then invited to an information meeting. The relatives are informed that they can withdraw their consent at any time. If consent is given, a medical doctor not involved in the trial but acting as trial guardian is asked to give consent as well. Written informed consent must be obtained from the patient himself or herself if he or she regains consciousness and decision-making capability during the trial period.

### Participants

Participants included in this trial must be admitted to the NICU at Rigshospitalet, Copenhagen, Denmark, with severe TBI, be at least 18 years old, and have a clinical presentation that does not exclude a later diagnosis of vegetative state or minimally conscious state or a GCS score of lower than 11 points during wake-up call, and stable intracranial pressure of less than 20 mm Hg for 24 h, and informed consent from the nearest relative and trial guardian must be in place. Patients with unstable fractures or other injuries that contraindicate mobilisation, patients with spinal cord injury, or patients without relevant informed consent are excluded from the trial.

### Time schedule

**Fig. 2:**
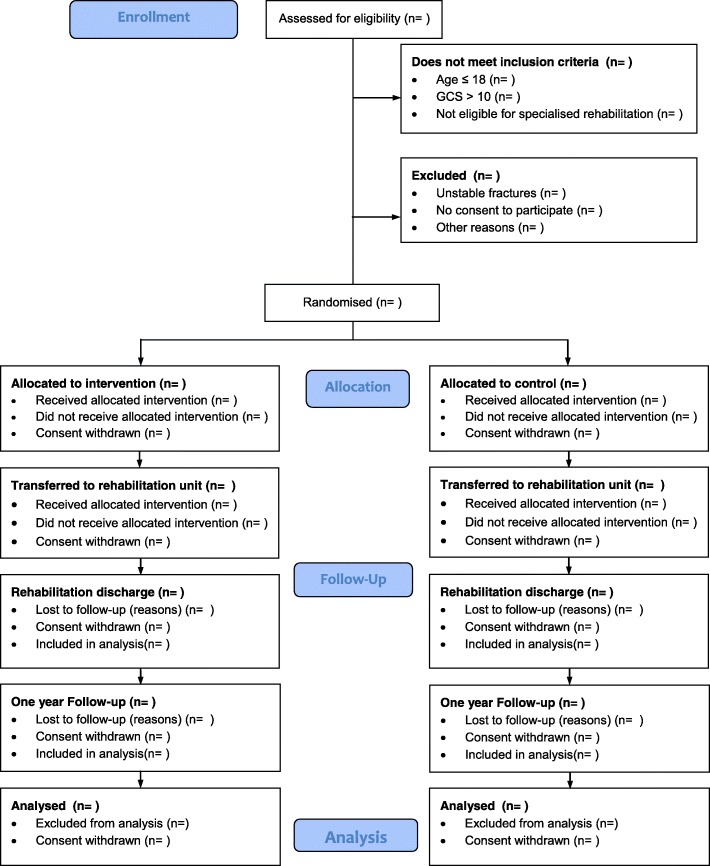
Trial flow diagram. Abbreviations: *GCS* Glasgow Coma Scale, *ICP* intracranial pressure

We will include patients until January 1, 2019 or until a maximum of 60 patients have been included, whichever occurs first. For a detailed flowchart on patient inclusion, please refer to Fig. 2. Data analysis will commence 3 months after (April 1, 2019). At this time, the 1-year follow-up will most likely not be complete for all patients. This variable will remain blinded until all data are gathered (January 1, 2020). A full statistical analysis plan will be developed before April 1, 2019.

### Early daily mobilisation (experimental intervention group)

In addition to standard care (see below), the experimental intervention group is subjected to an early and daily mobilisation protocol with HUT during their stay in the intensive care unit and throughout the early stages of rehabilitation. Mobilisation will be conducted using a tilt table with an integrated stepping device, which activates the venous pump and counteracts pooling of blood in the lower extremities (ERIGO^®^, Hocoma, Volketswil, Switzerland). The tilt-table intervention is applied once daily, 5 days per week for 4 weeks during the stay in the NICU. The duration of upright positioning is 20 min per session. Within each session, the patient will be moved to the tilt table and secured with straps and harness. The patient is then mobilised stepwise to 30°, 50°, and 70° HUT at 1-minute intervals while blood pressure (BP), heart rate (HR), respiratory rate, and peripheral oxygen saturation are closely monitored. Cerebral perfusion pressure (CPP) and intracranial pressure (ICP) are monitored as clinically indicated. If at any time our predetermined safety limits for BP, CPP, ICP, or HR (Table 1) are crossed, the patient is lowered to 0° tilt. When the values have returned within the safety limits, the procedure is resumed until the patient has been in the upright position for a maximum of 20 min or until a total duration of 40 min for the HUT session has been reached, whichever occurs first.

**Table 1:** Predetermined safety limits during head-up tilt

Absolute	Systolic/Diastolic BP	>80/50 mm Hg
	CPP	>50 mm Hg
	HR	<180 bpm
	ICP	<25 mm Hg
Relative	Permitted decrease from baseline systolic/diastolic BP	<30/15 mm Hg
	Permitted increase in HR from baseline	<30 bpm

Abbreviations: *BP* blood pressure, *CPP* cerebral perfusion pressure, *HR* heart rate, *ICP* intracranial pressure

If the patient is discharged from the NICU to the department of neurorehabilitation/TBI unit before the 4-week intervention period has ended, training will continue at the latter institution with a prespecified tilt-table protocol consisting of mobilisation twice a day on a similar tilt table. Patients who show functional improvement beyond the scope of tilt-table training (for example, are able to stand from a chair) before the trial period has ended will have their experimental intervention withdrawn and their final evaluation performed immediately hereafter; subsequently, the standard rehabilitation regimen will be continued.

Some patients will be discharged from the NICU to a temporary stay at another intensive care unit in the Capital Region of Copenhagen. In that case, the mobilisation and assessments will be continued using a standard tilt table (without the stepping device).

### Standard care (experimental and control groups)

The experimental and control groups receive standard rehabilitation as decided in collaboration between doctors, nurses, and physiotherapists and will be monitored during the trial. Only a small amount of time is used on mobilising the patient to either the edge of the bed or to a wheelchair whilst admitted to the NICU. The physiotherapists’ main focus is on respiratory function and positioning to avoid bedsores. The patients in the control group do not receive physical therapy on a daily basis.

### Trial duration

The trial intervention will consist of 4 weeks of mobilisation corresponding to 20 mobilisation sessions (Fig. 1). The patients included will be followed until end of in-hospital rehabilitation and again 1 year after injury.

### Data collection

Information on patient characteristics (age, sex, diagnosis, comorbidities, functional status prior to this injury, time since injury, and surgical procedures) is retrieved from the patient charts. For patients receiving the experimental intervention, the number of training sessions during the 4-week intervention period is recorded.

Outcomes are described below. There are two types of outcomes: feasibility outcomes and exploratory clinical outcomes.

### Feasibility outcomes

The primary objective of this trial is to assess feasibility. First, we will evaluate the number of patients we are able to include in the trial during the 2-year inclusion period, and a proportion of 60% or more of TBI patients who are eligible for the trial is acceptable. Second, we will evaluate the number of sessions applied in the experimental intervention group. The intervention will be considered to be feasible if at least 60% of the intended sessions (maximum of two per weekday in the trial for 4 weeks after randomisation, for a maximum of 20 sessions in total) are given to at least 52% of the patients in the intervention group. If a patient is transferred to another department and it is not possible to apply the intervention, we will count sessions as missing. If a patient dies, the number of applied and missing sessions is recorded at the time of death. Both feasibility outcomes are based on clinical judgement from the staff at the department and the trial investigators.

AEs, serious AEs (SAEs), ARs, serious ARs (SARs), and suspected unexpected SARs will be monitored during the trial counting the number of occurrences. Causality of AR will be assessed daily.

For a larger trial to be deemed feasible, both feasibility outcomes need to be attained, meaning that more than 60% of eligible patients will participate and at least 52% of the intervention group will receive more than 60% of the intended interventions.

### Exploratory clinical outcomes

The exploratory clinical outcomes are the CRS-R [20], the Early Functional Ability (EFA) [21], and the Functional Independence Measure (FIM) [22]. The CRS-R [20] evaluates changes in consciousness. It is hierarchically ordered and composed of six categories evaluating auditory, visual, motor, oromotor–verbal function, communication, and arousal. The scale ranges from 0 to 23 points, and a higher score indicates a higher level of function [20]. The evaluation will be carried out by two assessors who are experienced at using the scale. These assessors will be blinded to the patient’s treatment allocation. To obtain a complete evaluation of the patient’s progress, the EFA scale is included. The EFA scale is constructed to fill the evaluation gap between the GCS and FIM. The scale comprises 20 items, including measures of wakefulness, activities of daily living and cognitive abilities [21]; again, a higher score (range from 20 to 100 points) indicates a higher level of function. The FIM consists of 18 items highlighting motor function, ability to do activities of daily living and higher cognitive functions ranging from 18 to 126 points, and a higher score indicates a higher level of function. Scoring will be conducted by the staff at the two departments. It is not possible to blind these assessors to the randomisation procedure. The FIM has been thoroughly investigated in patients with TBI and has been shown to be valid and reliable and have established measures for detecting the minimal clinically important difference [23, 24]. The FIM was chosen as an outcome to track patient improvements over a long period of time. Owing to the initial low levels of consciousness in patients with severe TBI, combining the EFA and FIM has been recommended for a more complete assessment [25]. Preferably, the patient will be tested by CRS-R at the same time of day. The FIM and the EFA will be scored at the NICU by one tester with experience from the department of neurorehabilitation, who will gather necessary information from the multidisciplinary team treating the patient. Assessment of the patients at the department of neurorehabilitation will be performed by members of the clinical staff, who are experienced at using the two scales. The FIM, EFA, and CRS-R will be applied at baseline, at 4 weeks and 3 months after the baseline assessment, and at 1 year after the initial injury (Fig. 1).

Furthermore, length of stay at the two departments, time until tilt-table training is no longer relevant, and the duration of post-traumatic amnesia, as defined by time from injury and until the patient regains coherent day-to-day memory [26] are also registered. During the trial, the total amount of physical therapy sessions allocated to the patients is measured in both groups.

To address the haemodynamic changes during the transition from the supine position to HUT, we will measure non-invasive blood pressure by beat-to-beat photopletysmography and HR by ECG (ADInstruments, Oxford, UK), cerebral blood flow velocity (transcranial Doppler, Multi-Dop^®^ T digital, Compumedics Germany/DWL, Singen, Germany), and partial pressure of carbon dioxide in arterial blood (PaCO_2_) (ABL800, Radiometer, Copenhagen, Denmark). The HUT test will take place at baseline, after 2 weeks and after 4 weeks, or at the end of the intervention period. The data are used to investigate orthostatic tolerance and cerebral autoregulation as well as the patient’s baroreceptor sensitivity (beat-to-beat variation). Furthermore, ECG will be recorded continuously for 5 days, immediately after the patient has been included in the trial (ePatch, BioTelemetry Technology Aps, Hørsholm, Denmark).

### Statistical analyses

The primary feasibility outcome is the ratio between patients included and eligible patients. Eligible patients are those who fulfil the inclusion criteria of our trial. For example, if the number of randomly assigned participants is 44 out of 60 eligible patients, then the proportion will be 0.73 with a 95% confidence interval (CI) between 0.60 and 0.84. A proportion of 0.60 (the lower CI of the proportion) or more randomly assigned patients will be acceptable for a future larger-scale trial. We strive for having as large a proportion of eligible patients as participants to make the latter as representative of the former as possible and have arbitrarily set the acceptable lower 95% CI to be 60% or above. We will include a maximum of 60 participants or as many as possible during the 24-month recruitment period.

The second feasibility outcome is defined as the number of HUT sessions applied during the 4-week intervention period. In our clinical judgement, we believe that it is satisfactory to be able to apply more than 60% of the daily HUT sessions on weekdays for more than 70% or at least 52% (the lower CI of the proportion) of the patients. Since a maximum of 30 patients will be randomly assigned to the intervention, a binominal distribution is calculated from the proportion of 70%, which gives a lower 95% CI of 52%.

The number of patients with at least one AE or SAE during the intervention period will be analysed as exploratory feasibility outcomes using logistic regression adjusted for the protocol-specified stratification variable. Moreover, we will compare the proportions and severity in the two intervention groups.

Baseline data will be used to describe the population. Data will be analysed by using SAS Enterprise version 7.11 (SAS Institute Inc., Cary, NC, USA). A binominal distribution will be used to calculate the 95% CI for our primary feasibility outcome as the proportion of randomly assigned patients from the eligible patients.

The clinical exploratory outcomes will not undergo traditional statistical testing, as this is a small feasibility trial with large risks of random errors. However, in order to test the feasibility of the analyses and for exploratory purposes, outcomes will be analysed and *P* values will be presented. *P* values of any size will not be interpreted as “significant”.

The CRS-R, FIM, and EFA as well as the physiological measures of mean arterial pressure, HR, cerebral blood flow, and the dynamic autoregulation index contain multiple measurement points and will be analysed accordingly with analysis of variance or other linear regression models for repeated measures. Missing data will be treated with the multiple imputation method.

Dynamic cerebral blood flow autoregulation is analysed as the ratio between mean arterial pressure and cerebral blood flow velocity. For this, a Pearson correlation coefficient of 30 mean values of mean arterial pressure and cerebral blood flow each consisting of 10 s of measurements are correlated in the supine position and during maximum HUT [12, 27]. This gives two values of the so-called Mx index per tilt test. Baroreceptor regulation is assessed by using data from the ECG waves to conduct a power spectral analysis of the RR intervals. The purpose is to analyse the low-frequency content (0.05 to 0.15 Hz), which is assumed to reflect the baroreceptor activity, as well as the high-frequency content (0.15 to 0.35 Hz), which is related mainly to parasympathetic activity [28].

All analyses will be intention-to-treat using multiple imputations to account for missing data as described by Jakobsen et al. [29]. Analyses will be conducted blinded with the two intervention groups coded as, for example, 0 and 1. After the drawing of conclusions, the blinding will be broken.

## Discussion

Early physical rehabilitation has previously been associated with improved outcome in patients with TBI in a cohort study [13]. The pilot study published by Frazzitta et al. showed promising results when starting physical rehabilitation early in 31 patients with ABI, of whom 12 were affected by TBI [30]. Nevertheless, there is a lack of studies investigating the causal relationship between early physical rehabilitation and long-term outcome. Andelic et al. conducted a quasi-randomised cohort study on the effects of early rehabilitation at the intensive care unit in patients with TBI [13]. Although the consistency of the rehabilitation paradigm was unspecified, they did observe a benefit of this intervention as measured by the Glasgow Outcome Scale Extended and the Disability Rating Scale after 12 months [13]. This trial intends to lay the foundation for a larger-scale multicentre randomised clinical trial, investigating whether the patients are able to tolerate HUT, whether the intervention is practically feasible, and whether the outcomes are improved. A trial comparing short- and long-term functional outcomes after standard care compared with early mobilisation should also assess the effects of mobilisation on haemodynamic regulation which has previously been associated with poor outcome or death [11, 12], in an attempt to identify potential predictors of long-term recovery. The trial is designed aiming for a low risk of bias using centralised randomisation, blinded outcome assessors where possible, and blinded statistical analyses [31–33]. However, it is not possible to blind the patients or care givers, which may lead to risk of bias. Furthermore, given the small sample size and the heterogeneous trial population, any differences we find between groups may be due to selection bias or random errors or both [33–35]. Therefore, any result should be interpreted with great caution.

Investigating AEs and ARs is with limitations. Whether or not there is a direct causal relation between an incidence and an AE and the intervention can in many ways be subjective and hard to determine. Nevertheless, we feel confident that the experienced staff can provide support in informing when in doubt. We believe it is important to do this feasibility trial as a randomised clinical trial since it is likely to affect the decision of entering the trial. Whether the mobilisation intervention is feasible could have been answered in a classic observational study.

It is difficult to provide sufficient evidence for the general assumptions presented in this protocol that longer periods of bed rest may influence the baroreceptor sensitivity and that early mobilisation may re-establish it. Using HR variability to assess regulation of the autonomic nerve system has been the subject of debate but is a relatively simple, non-invasive tool, even though more sophisticated and invasive measures could be used for measuring sympathetic nerve activity, such as direct recording of single-fibre muscle sympathetic nerve activity.

If a larger multicentre randomised clinical trial is deemed feasible, the data gathered in the present trial should be of great use. The required sample size of a larger randomised clinical trial shall be calculated on the basis of the data from the likely effects from the present trial as well as evidence from updated systematic reviews of randomised clinical trials. Moreover, the financial estimates of conducting a larger trial will be clearer from estimates of time consumption based on the present feasibility trial.

## Trial status

Enrolment commenced on January 2, 2017. At present, 34 patients have been randomly assigned. We will continue including patients until January 1, 2019 or until 60 patients are included, whichever occurs first, and will complete the last 1-year follow-up assessment in December 2019.

## Additional file

Additional file 1: SPIRIT (Standard Protocol Items: Recommendations for Interventional Trials) 2013 Checklist: Recommended items to address in a clinical trial protocol and related documents*. (DOC 122 kb)

## Abbreviations

ABI: Acquired brain injury
AE: Adverse event
AR: Adverse reaction
BP: Blood pressure
CI: Confidence interval
CPP: Cerebral perfusion pressure
CRS-R: Coma Recovery Scale–Revised
ECG: Electrocardiography
EFA: Early Functional Ability
FIM: Functional Independence Measure
GCS: Glasgow Coma Scale
HR: Heart rate
HUT: Head-up tilt
ICP: Intracranial pressure
NICU: Neurointensive care unit
SAE: Severe adverse event
SAR: Severe adverse reaction
TBI: Traumatic brain injury
